# Overexpression of Human-Derived DNMT3A Induced Intergenerational Inheritance of Active DNA Methylation Changes in Rat Sperm

**DOI:** 10.3389/fgene.2017.00207

**Published:** 2017-12-12

**Authors:** Xiaoguo Zheng, Zhenhua Li, Guishuan Wang, Zhengzheng Li, Ajuan Liang, Hanshu Wang, Yubing Dai, Xingxu Huang, Xuejin Chen, Yuanwu Ma, Fei Sun

**Affiliations:** ^1^International Peace Maternity & Child Health Hospital, Institute of Embryo-Fetal Original Adult Disease, School of Medicine, Shanghai Jiao Tong University, Shanghai, China; ^2^Center for Reproductive Medicine, School of Medicine, Renji Hospital, Shanghai Jiao Tong University, Shanghai, China; ^3^School of Life Science and Technology, ShanghaiTech University, Shanghai, China; ^4^Department of Laboratory Animal Science, Shanghai Jiao Tong University School of Medicine, Shanghai, China; ^5^Key Laboratory of Human Disease Comparative Medicine, Institute of Laboratory Animal Science, Chinese Academy of Medical Sciences, Beijing, China

**Keywords:** human-derived *DNMT3A*, DNA methylation, sperm, rat, intergenerational inheritance

## Abstract

DNA methylation is the major focus of studies on paternal epigenetic inheritance in mammals, but most previous studies about inheritable DNA methylation changes are passively induced by environmental factors. However, it is unclear whether the active changes mediated by variations in DNA methyltransferase activity are heritable. Here, we established human-derived *DNMT3A* (*hDNMT3A)* transgenic rats to study the effect of *hDNMT3A* overexpression on the DNA methylation pattern of rat sperm and to investigate whether this actively altered DNA methylation status is inheritable. Our results revealed that *hDNMT3A* was overexpressed in the testis of transgenic rats and induced genome-wide alterations in the DNA methylation pattern of rat sperm. Among 5438 reliable loci identified with 64 primer-pair combinations using a methylation-sensitive amplification polymorphism method, 28.01% showed altered amplified band types. Among these amplicons altered loci, 68.42% showed an altered DNA methylation status in the offspring of transgenic rats compared with wild-type rats. Further analysis based on loci which had identical DNA methylation status in all three biological replicates revealed that overexpression of *hDNMT3A* in paternal testis induced hypermethylation in sperm of both genotype-negative and genotype-positive offspring. Among the differentially methylated loci, 34.26% occurred in both positive and negative offspring of transgenic rats, indicating intergenerational inheritance of active DNA methylation changes in the absence of *hDNM3A* transmission. Furthermore, 75.07% of the inheritable loci were hyper-methylated while the remaining were hypomethylated. Distribution analysis revealed that the DNA methylation variations mainly occurred in introns and intergenic regions. Functional analysis revealed that genes related to differentially methylated loci were involved in a wide range of functions. Finally, this study demonstrated that active DNA methylation changes induced by *hDNMT3A* expression were intergenerationally inherited by offspring without transmission of the transgene, which provided evidence for the transmission of active endogenous-factors-induced epigenetic variations.

## Introduction

DNA methylation is a conserved epigenetic marker that generally involves the addition of a methyl group to a cytosine base to form 5-methylcytosine (5 mC). It is associated with many important biological processes in mammals, including regulation of gene expression, chromatin reorganization, X chromosome inactivation, genomic imprinting and epigenetic reprogramming (Bird, 2002; Messerschmidt et al., 2014; Cotton et al., 2015). Sequencing at single-base resolution reveals that DNA methylation occurs predominantly at CpG dinucleotides, and hyper-methylation usually occurs in centromeres, pericentromeric regions, transposons and other repetitive elements, while hypomethylation occurs within gene promoter regions (Lister et al., 2009; Habibi et al., 2013). However, non-CG methylation, such as CHG and CHH (where H = C, T, or A) methylation, is also present at detectable levels, especially in embryonic stem cells and oocytes (Ramsahoye et al., 2000; Shirane et al., 2013). Previous studies have revealed that DNA methylation dynamically changes during embryo development in mammals (Smallwood et al., 2011; Guibert et al., 2012; Smith et al., 2012). Aberrant DNA methylation patterns in organisms can result in multiple diseases and even embryonic lethality. For example, abnormal DNA methylation status in the paternal imprinted gene *H19* induced male infertility and deficient offspring embryonic development (Ibala-Romdhane et al., 2011). Fragile X syndrome, various cancers and Prader-Willi/Angelman syndrome in *in-vitro*-conceived children were associated with aberrant DNA methylation status in imprinting control regions (Alisch et al., 2013; Bergman and Cedar, 2013; Cotton et al., 2015). These studies suggest that DNA methylation plays important roles in gametogenesis and embryogenesis in mammals.

Recent studies have demonstrated that exposure to environmental factors, such as toxicants, stress or dietary compounds, can induce DNA methylation changes in germ cells and induce various abnormalities in mammalian offspring (Park et al., 2012; Martinez-Arguelles et al., 2013; Ost et al., 2014). Moreover, some of these epigenetic variations and the accompanying acquired characteristics can be transmitted to offspring intergenerationally and/or transgenerationally (Carone et al., 2010; Radford et al., 2014; Wei et al., 2014). Thus, these epigenetic inheritance systems provide a potential mechanism by which parents can transfer information about the environment they experienced to their offspring (Wei et al., 2014). Nevertheless, the inheritance of epigenetic marks indicates that these systems overrode the reprogramming process by which the offspring embryos undo any potentially deleterious bookmarking that their parents' lifetime experience may have imposed. Therefore, epigenetic inheritance and epigenetic reprogramming are two antagonistic processes. The underlying mechanisms responsible for epigenetic inheritance of environmentally induced phenotypes remain unknown (Radford et al., 2014). Furthermore, the heritable DNA methylation changes mentioned above are induced by environmental factors; in other words, these DNA methylation changes are passive changes. Further studies should focus on whether active DNA methylation changes, which are mediated by altered activities of DNA methyltransferases and enzymes that related to demethylation, are heritable.

In mammals, DNA methylation patterns are established early in development through a highly orchestrated process that involves genome-wide demethylation and *de novo* methylation (He et al., 2011). In mice, two waves of genome-wide DNA demethylation, followed by two waves of genome-wide DNA remethylation, occur during primordial germ cell (PGC) development and early embryogenesis (Wang et al., 2014). These two rounds of DNA methylation erasure—parental genomes undergo extensive DNA demethylation via both active and passive mechanisms—leave little chance for inheritance of DNA methylation changes that are environmentally induced. Otherwise, two rounds of DNA methylation establishment—parental genomes undergo extensive DNA remethylation via active mechanisms—affect gametogenesis and early embryogenesis. During the epigenetic reprogramming process, DNA demethylases and methyltransferases are responsible for DNA methylation erasure and re-establishment, respectively. In mammals, there are four active DNA methyltransferases, including the well-characterized *DNMT1, DNMT3A*, and *DNMT3B* and the newly discovered *DNMT3C* (Law and Jacobsen, 2010; Barau et al., 2016). DNA methylation patterns are established by *DNMT3A, DNMT3B*, and *DNMT3C* through *de novo* methylation pathways and maintained by the maintenance methyltransferase *DNMT1* during DNA replication (Law and Jacobsen, 2010). *DNMT3A* and *DNMT3B* were reported to be primarily responsible for the genome-wide *de novo* methylation during the period of embryo implantation and the period of PGC development to establish a new methylation pattern in the offspring embryos and germlines, respectively (Okano et al., 1999). Although the functional deficiency of DNA methyltransferases leads to abnormal embryo development and even embryonic lethality in mammals [26], the impact of *DNMTs* overexpression on the DNA methylation pattern of germ cells is still unclear.

Overexpression of the human-derived histone 3 lysine 4 (H3K4) demethylase KDM1A (also known as LSD1) during spermatogenesis in mouse testis reduced H3K4 dimethylation and altered RNA content in mouse sperm (Siklenka et al., 2015). These active epigenetic changes reduced survival and increased abnormal development of the offspring; the altered histone modification patterns and acquired characteristics were intergenerationally and transgenerationally inherited by the progeny (Siklenka et al., 2015). In this study, we established humanized rats to study the impact of human-derived *DNMT3A* (*hDNMT3A*) overexpression on the DNA methylation pattern of rat sperm and to investigate whether the DNA methylation changes induced by *hDNMT3A* overexpression in paternal sperm can be transmitted to the offspring in the absence of *hDNMT3A*.

## Materials and methods

### *hDNMT3A* transgenic rats

All studies involving animals were conducted under the approval of the Institutional Animal Care Committee of Shanghai Institute of Biochemistry and Cell Biology. The *hDNMT3A* transgenic rats were established using zygote microinjection of a bacterial artificial chromosome (BAC). The RP11-159D24 BAC (NCBI-ID:223335, *Homo sapiens*, GRCh38.p2: Chr. 2: 25,202,642-25,410,145, total length: 207,504 bp) containing DNMT3A (GRCh38.p2: 25,232,961,25,342,590, total length: 109,630 bp) was injected into rat zygotes (*Rattus norvegicus*, Sprague-Dawley, SD). The offspring were identified with 8 PCR primer-pairs that were specific for the RP11-159D24 sequence to detect positive transgenic ones, and a wild-type rat was used as negative control (Supplemental Table 1).

### Using qPCR to analyse the copy number of *hDNMT3A*

Then, we performed qPCR to identify the copy numbers of *hDNMT3A* in male genotype-positive transgenic rats (founder). The reaction volume contained 20 ng genomic DNA, 10 μl 2x Trans Start Top Green qPCR SuperMix (TransGen, China), 1.6 μl forward primer (2.5 μM), 1.6 μl reverse primer (2.5 μM), and 0.4 μl Passive Reference Dye II (50x), and ddH_2_O was added to a final volume of 20 μl. The PCR procedure was performed as follows: 95°C 30 s; 95°C 5 s, and 60°C 34 s for 40 cycles followed by a dissociation curve on an ABI Prism 7,500 Real Time Thermal Cycler (Applied Biosystems, Life Science, U.S.). Three biological replicates and three technical replicates were set. In this study, gamma-glutamyltranspeptidase 1 (γGgt1, Gene ID: 116568) was used as internal reference as it was reported as a single copy gene in rat (Pawlak et al., 1988). The primers used in this study are as follows: γGgt1-F (forward): 5′-TCCTAGTGAATGCTCTTGTGCT-3′, γGgt1-R (reverse): 5′-TGAAGTCATCC-ATCTCGTCATT-3′; *hDNMT3A*-F: 5′-GCTATTCGCCTTGC-TATTG-3′, *hDNMT3A*-R: 5′-AGAA-AACTACCTGGAGATGGCC-3′. *hDNMT3A* single-copy-insertion transgenic male rat (founder) was selected to mate with wild-type female SD-rat, and their genotype-positive offspring (F1 generation) were mated with wild-type female SD-rat to obtain F2 generation.

### RT-qPCR analysis of the *hDNMT3A* and rDNMT3A expression patterns

Total RNA was extracted from three tissues (liver, brain, and testis) of wild-type, positive and negative transgenic adult rats (F1 generation, body weight >280 g) using TransZol Up (TransGen, China). The quality and concentration of total RNA was detected by a NanoDrop2000 (Thermo Fisher Scientific, U.S.). Then, 1 μg RNA was used to synthesize the cDNA according to the instructions of the PrimeScript™ RT reagent Kit with gDNA Eraser (TaKaRa, Japan). The reaction volume contained 0.5 μl synthesized cDNA, 10 μl 2x Trans Start Top Green qPCR SuperMix (TransGen, China), 1.6 μl forward primer (2.5 μM), 1.6 μl reverse primer (2.5 μM), 0.4 μl Passive Reference Dye II (50x) and ddH_2_O to a final volume of 20 μl. The PCR procedure was performed as above. Three biological replicates and three technical replicates were set. The results were normalized by the rat house-keeping gene *Gapdh*. The primers used in this study were listed as follows: *hDNMT3A*-F: 5′-AGTTAGCAGCAGGGAGACGA-3′, *hDNMT3A*-R: 5′-AAGAGGTAACAGCGGCTTCTA-3′; *rDnmt3a*-F: 5′-GAAACCCAGAAAGAGCACAAC-3′, *rDnmt3a*-R: 5′-CTTACAGTTCT-GGCACATTCCA-3′; *rGapdh*-F: 5′-GGCAAGTTCAATG-GCACAGT-3′, *rGapdh*-R: 5′-TGGTGAAGACGCCAGTAGACTC-3′.

### Sperm genomic DNA extraction

The cauda epididymidis of genotype-positive and negative rats of F2 generation were separated. Then, the tissues were gently cut and incubated at 37°C and 5% CO_2_ for 20 min in 500 μl M2 medium (Sigma-Aldrich, Merck, U.S.) to ensure that the sperm was completely liberated. Approximately 450 μl supernatant was collected into an Eppendorf tube. After a 5 min incubation, 400 μl supernatant was collected into a new tube, following by 500 g centrifugation for 5 min. After centrifugation, the supernatant was discarded, and the sperm was resuspended with 500 μl 1x PBS solution, following by centrifugation at 500 g for 5 min. This step was conducted three times to remove the seminal plasma contaminants. Sperm genomic DNA was extracted using an EasyPure Genomic DNA Kit (TransGen, China) with addition of dithiothreitol (DTT, 40 mM) to disturb the disulfide bonds. The sperm DNA of genotype-positive and negative rats were used for DNA methylation detection.

### Methylation-sensitive amplification polymorphism (MSAP) assay

The DNA methylation pattern of rat sperm was detected using techniques of methylation sensitive restriction enzyme digestion and PCR amplification. The MSAP analysis was performed as described in our previous study (Zheng et al., 2013). Three biological replicates for each genotype (wild-type, genotype-negative and genotype positive F2 male rats) were used in this study. In brief, two digestion reactions were set up at the same time for each sperm genomic DNA sample. One reaction contained both *EcoR* I and *Msp* I (New England Biolabs, U.S.), while another reaction contained both *EcoR* I and *Hpa* II (New England Biolabs, NEB, U.S.). The digestion volume contained 300 ng genomic DNA, 10 U *EcoR* I, 10 U *Msp* I (or *Hpa* II), 2 μl Cutsmart buffer (NEB, U.S.), and ddH_2_O to a final volume of 20 μl. After incubation at 37°C for 6 h, 5 μl digestion product was assessed with 0.5% agarose gels to ensure that the genomic DNA was completely digested (Supplemental Figure 1). The ligation reaction contained 15 μl digestion product, 5 pmol *EcoR* I adapter (Supplemental Table 2), 50 pmol *Hpa* II/*Msp* I (H/M) adapter (Supplemental Table 2), 1.5 U T4 ligase (NEB, U.S.), 3 μl ATP (NEB, U.S.), 1.5 μl Cutsmart buffer (NEB, U.S.), and ddH_2_O to a final volume of 30 μl. After incubation at 16°C overnight (8–12 h), the reaction was denatured at 65°C for 20 min to inactivate the enzymes. The ligation product was diluted 20-fold before pre-amplification. The pre-amplification reaction contained 5 μl 10× PCR buffer, 4 μl dNTPs (2.5 mM), 5 U rTaq (TaKaRa, Japan), 5 μl diluted ligation product, 2 μl E1 primer (10 μM, Supplemental Table 2), 2 μl HM1 primer (10 μM, Supplemental Table 2), and ddH_2_O to a final volume of 50 μl. The PCR program was as follows: 94°C 5 min; 94°C 30 s, 56°C 30 s, and 72°C 1 min for 29 cycles, 72°C 10 min and 4°C hold. The selective amplification reaction contained 2 μl 10× PCR buffer, 1.6 μl dNTPs (2.5 mM), 1 U rTaq (TaKkaRa, China), 1 μl pre-amplification product, 1 μl *EcoR* I selective primers (10 μM, E02-E05, Supplemental Table 2) and 1 μl H/M-selective primers (10 μM, HM01-HM16, Supplemental Table 2). All *EcoR* I-selective primers were modified with 5′-FAM. The PCR program was as follows: 94°C 5 min; 94°C 30 s, 65°C 30 s, and 72°C for 1 min, decrease in the annealing temperature by 0.7°C per cycle during 12 cycles, and then 24 cycles of 94°C for 30 s, 56°C for 30 s, and 72°C for 1 min with a final extension of 10 min at 72°C. The selective amplification product was detected by capillary electrophoresis (3730xl DNA analyzer, Applied Biosystems, U.S.) and analyzed by GeneMapper 4.0 (Applied Biosystems, U.S.). Only the bands that had a signal height more than 1,000, a fragment length longer than 50 bp but shorter than 500 bp, and the same pattern in at least two of three replicates were used for subsequent analysis (Supplemental Figure 2). To isolate the differentially methylated fragments, we conducted 6% polyacrylamide gel electrophoresis and visualized the bands via silver staining (Supplemental Figure 3).

### MSAP data analysis

**Figure 1:**
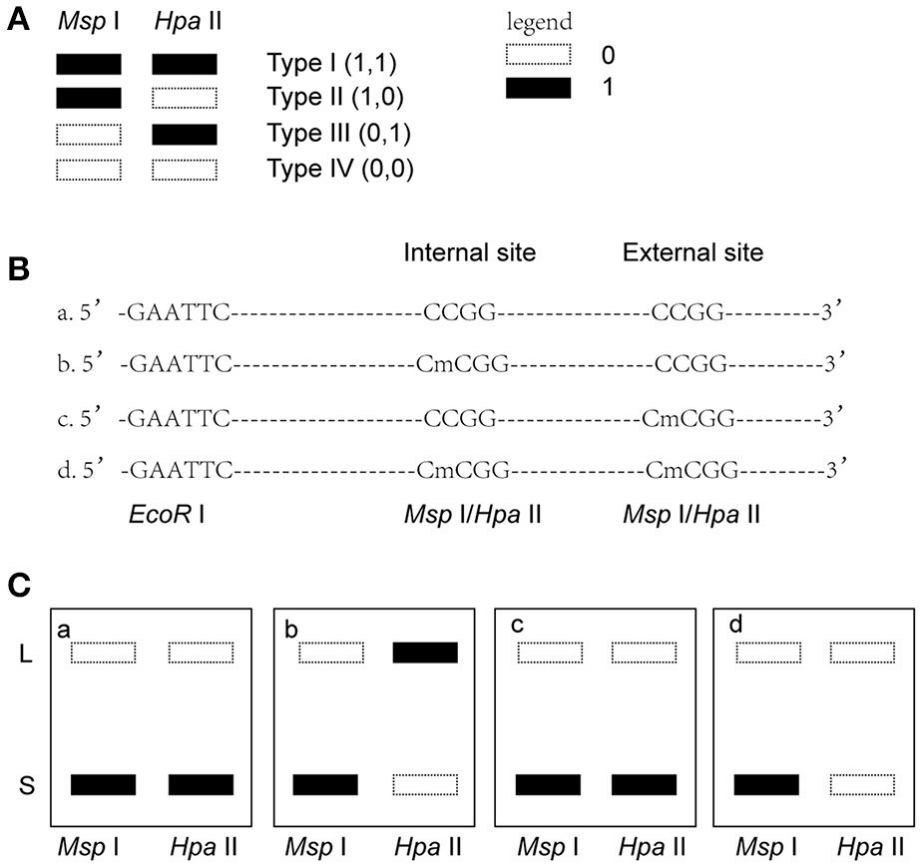
Analysis of different band types in this study. **(A)** Different band types in this study. Black bar represented presence of amplified fragments and was recorded as 1 while white bar represented absence of amplified fragments and was recorded as 0. Type I (1,1) represented unmethylated loci; Type II (1,0) represented full-methylated loci; Type III (0,1) represented hemimethylated loci in plants, but in mammals they would be occurred due to an internal CmCGG; Type IV (0,0) represented hyper-methylated loci in plants, but in mammals they would be occurred due to the comparison with type III bands or due to the loss of CCGG sequence. **(B)** Four possible DNA methylation patterns of two adjoining CCGG sequences. **(C)** Corresponding band types of four possible DNA methylation patterns. L (long fragment) represented that the internal CCGG was not digested by *Msp* I or *Hpa* II, but the external CCGG was digested and amplified with corresponding selective primer pair. S (short fragment) represented that the internal CCGG was digested by *Msp* I or *Hpa* II and amplified with corresponding selective primer pair. The type III (1,0) bands only occurred in case b, and corresponding band types of the other lanes were type IV (0,0) (a,c,d).

*Msp* I and *Hpa* II are isoschizomers and recognized the same sequence (5′-CCGG-3′), but they showed different sensitivity to the methylation status. Bands that were present in both the *EcoR* I/*Msp* I lane and *EcoR* I/*Hpa* II lane were recorded as type I (1,1), and represented unmethylated loci, i.e., both two DNA strands were unmethylated (Figure 1A, Supplemental Figures 2, 3). Bands that were present in the *EcoR* I/*Msp* I lane but not in the *EcoR* I/*Hpa* II lane were recorded as type II (1,0), and represented fully-methylated loci, i.e., both two DNA strands were methylated (Figure 1A, Supplemental Figures 2, 3). Bands present in the *EcoR* I/*Hpa* II lane but not in *EcoR* I/*Msp* I lane were recorded as type III (0,1) (Figure 1A, Supplemental Figures 2, 3). This band type represented hemi-methylated loci (mCCGG) in plants, but in vertebrate DNA methylation was almost exclusively in CG dinucleotide. Thus, we considered that the loci with this band type would be due to an internal CmCGG site (Figures 1B,C, Supplemental Figures 2, 3; Fulnecek and Kovarik, 2014). In addition, the loci which cut by neither enzyme were recorded as type IV (0.0) (Figure 1A). They were recognized by comparison with the other lanes (Figure 1C). These loci would be due to the comparison with type III bands or due to the loss of CCGG sequence (Figure 1C, Supplemental Figures 2, 3; Fulnecek and Kovarik, 2014). Differentially methylated loci were analyzed according to Table 1. In brief, the emergences of new 5′-CmCGG-3′ sequences and the loci that the amplified bands changed from type I (1,1) to type II (1,0) were considered as hypermethylation events while the loss of 5′-CmCGG-3′ sequences and the loci that the amplified bands changed from type II (1,0) to type I (0,0) were considered as hypomethylation events, i.e., the gain of mC was considered as hypermethylation while the loss of mC was considered as hypomethylation (Table 1). Only the bands that had the identical DNA methylation status in all three replicates were analyzed for further studies of transmission of differentially methylated loci. The DNA methylation ratio (%) of each sample was calculated by the following formula: type II/(type I + type II) ^*^100%. We used *t*-test to analyze the significant differences in DNA methylation levels among different genotypes, and the *p*-value was set as 0.05.

**Table 1:** The model of analyzing the differentially amplified loci.

**Changes in band types**	**Sequence change**	**Interpretation**	**Methylation variation**
Type IV (0,0) → Type I (1,1)	New CCGG	Mut.	Unchange
Type IV (0,0) → Type II (1,0)	New CmCGG	Mut. and epimut.	Hypermethylation
Type IV (0,0) → Type III (0,1)	From a, c, or d to b (Figure 1C)	Ambiguous	Unchange^*^
Type I (1,1) → Type IV (0,0)	CCGG loss	Mut.	Unchange
Type I (1,1) → Type II (1,0)	CCGG → CmCGG	Epimut.	Hypermethylation
Type I (1,1) → Type III (0,1)	New internal CmCGG	Mut. and epimut.	Hypermethylation
Type II (1,0) → Type IV (0,0)	CmCGG loss	Mut. and epimut.	Hypomethylation
Type II (1,0) → Type I (1,1)	CmCGG → CCGG	Epimut.	Hypomethylation
Type II (1,0) → Type III (0,1)	CmCGG → CCGG and new internal CmCGG	Mut. and epimut.	Unchange
Type III (0,1) → Type IV (0,0)	From b to a, c, or d (Figure 1C)	Ambiguous	Unchange^*^
Type III (0,1) → Type I (1,1)	Internal CmCGG loss	Mut. and epimut.	Hypomethylation
Type III (0,1) → Type II (1,0)	(CCGG → CmCGG) and internal CmCGG loss	Mut. and epimut.	Unchange

* *The DNA methylation variations in such cases could not be defined. Thus, we considered that they were unchanged in DNA methylation level*.

*Mut., Mutation (sequence mutation); Epimut., Epimutation (DNA methylation variation)*.

### Sequencing the differentially amplified loci (DAL)

Differentially amplified fragments were isolated from 6% polyacrylamide gel (Supplemental Figure 3) and re-amplified with corresponding selective primer-pair combinations. Then, the purified DNA was sequenced and aligned to the reference genome. GO (Gene Ontology) analysis was performed to annotate the functions of genes related to DALs (http://www.geneontology.org/) (Mi et al., 2013).

### Bisulfite sequencing PCR (BSP) for validation of MSAP

BSP is the gold standard method to analyze DNA methylation pattern. After bisulfite conversion, unmethylated cytosine was converted to uracil, while methylated cytosine or hydroxymethyl-cytosine was unchanged. After PCR, the unmethylated cytosine changed to thymine. The DNA methylation status of targeted cytosine could be determined by comparing the PCR product with the reference sequence. For validation of the results of MSAP assays, the DALs of #23 and #52 (Supplemental Table 3) was analyzed by BSP. Briefly, 2 μg sperm genomic DNA was converted and purified according to the instructions of the EpiMark bisulfite conversion kit (NEB, U.S.). The converted product was dissolved with 30 μl ddH_2_O. The PCR reaction contained 10 μl 5x EpiMark Hot Start Taq Reaction Buffer (NEB, U.S.), 1 μl dNTPs (10 mM, TaKaRa, Japan), 1 μl forward primer (10 μM), 1 μl reverse primer (10 μM), 0.25 μl EpiMark Hot Start Taq DNA Polymerase (NEB, U.S.), 6 μl converted genomic DNA, and ddH_2_O to a final volume of 50 μl. #23-F primer: 5′-TTTTTTAGTTTTAAGGGTTAGATTAGGATAGTTG-3′, #23-R primer: 5′-AACTACACTTC-AACATCCCCATAAAC-3′; #52-F primer: 5′-TATAGATTTTTTGGGGAGGTAGATTTTGAG-GTTAATAG-3′, #52-R primer: 5′-ACRAAAAAAAACCACACAATAAAAAACAAC-3′. The PCR procedure was as follows: 95°C 30 s; 95°C 30 s, 45°C 30 s, and 68°C 30 s for 45 cycles; 68°C 5 min and 4°C hold. The PCR product was purified with an EasyPure Quick Gel Extraction Kit (TransGene, China) and subsequently cloned into the pEasy-T1 vector (TransGene, China). Then, individual clones for each sample were sequenced to analyze the DNA methylation pattern of the targeted cytosine.

## Results

### *hDNMT3A* is expressed in the three tested tissues of the transgenic rats

**Figure 2:**
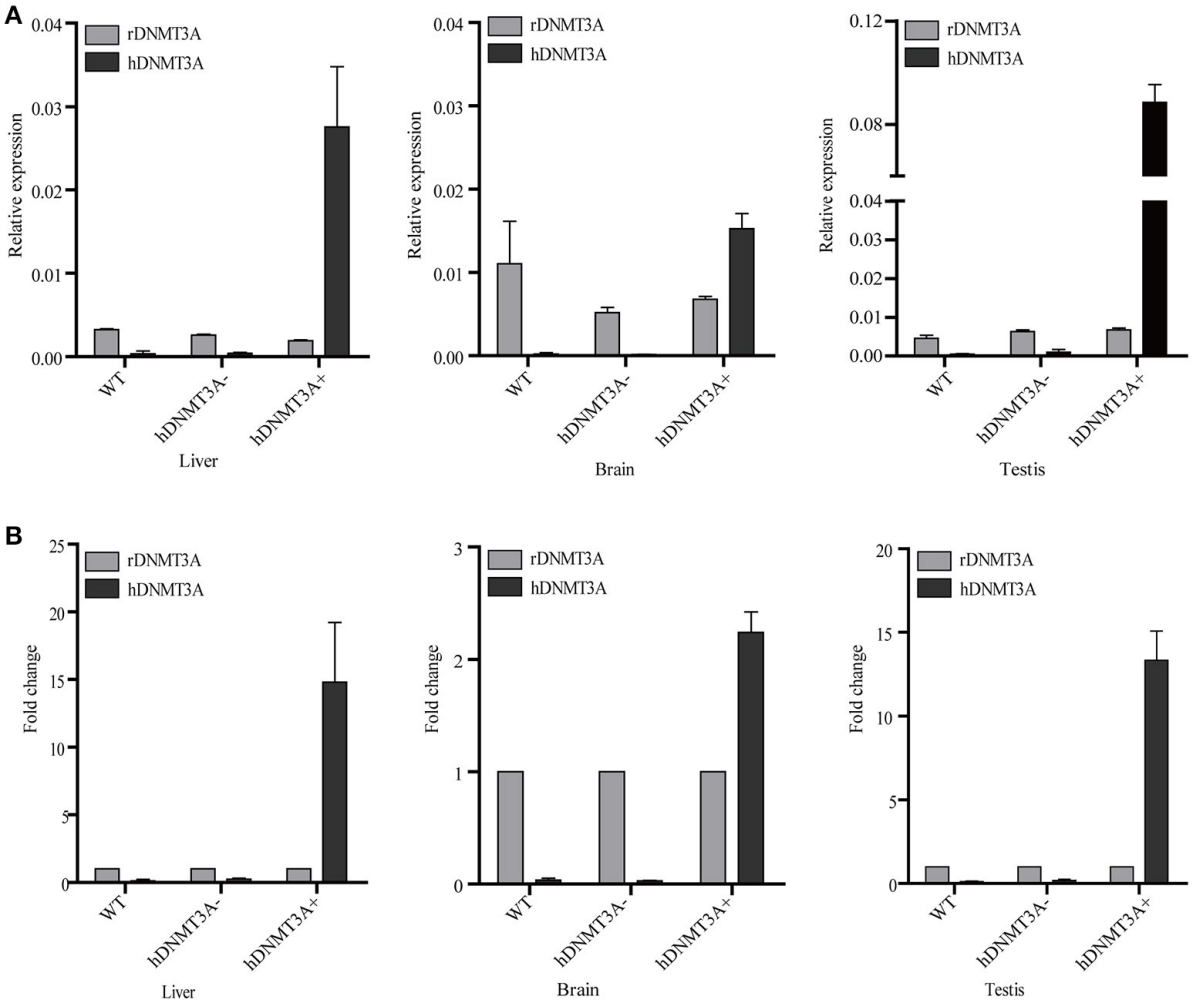
The expression pattern of *hDNMT3A* and rDNMT3A in liver, brain and testis of wild-type, negative and positive transgenic rats (F1 generation). **(A)** Relative expression levels of *hDNMT3A* and rDNMT3A to rGAPDH. **(B)** Relative expression of *hDNMT3A* to rDNMT3A.

We performed RT-qPCR to analyze the expression pattern of both *hDNMT3A* and rDNMT3A in three tissues of transgenic rats (F1 generation). The results revealed that *hDNMT3A* was overexpressed in all three tissues of transgenic progenies, especially in the testis, compared with rDNMT3A (Figure 2A). In liver of the *hDNMT3A*^+^ rats, the expression level of *hDNMT3A* was 14 times higher than that of the rDNMT3A (Figure 2B). In brain and testis, the numbers were 2.5 times and 13 times, respectively (Figure 2B). In WT and *hDNMT3A*^−^ rats, *hDNMT3A* mRNA was rarely detected in all three tissues.

### DNA methylation patterns in sperm of both hDNMT3A^−^ and hDNMT3A^+^ rats

**Figure 3:**
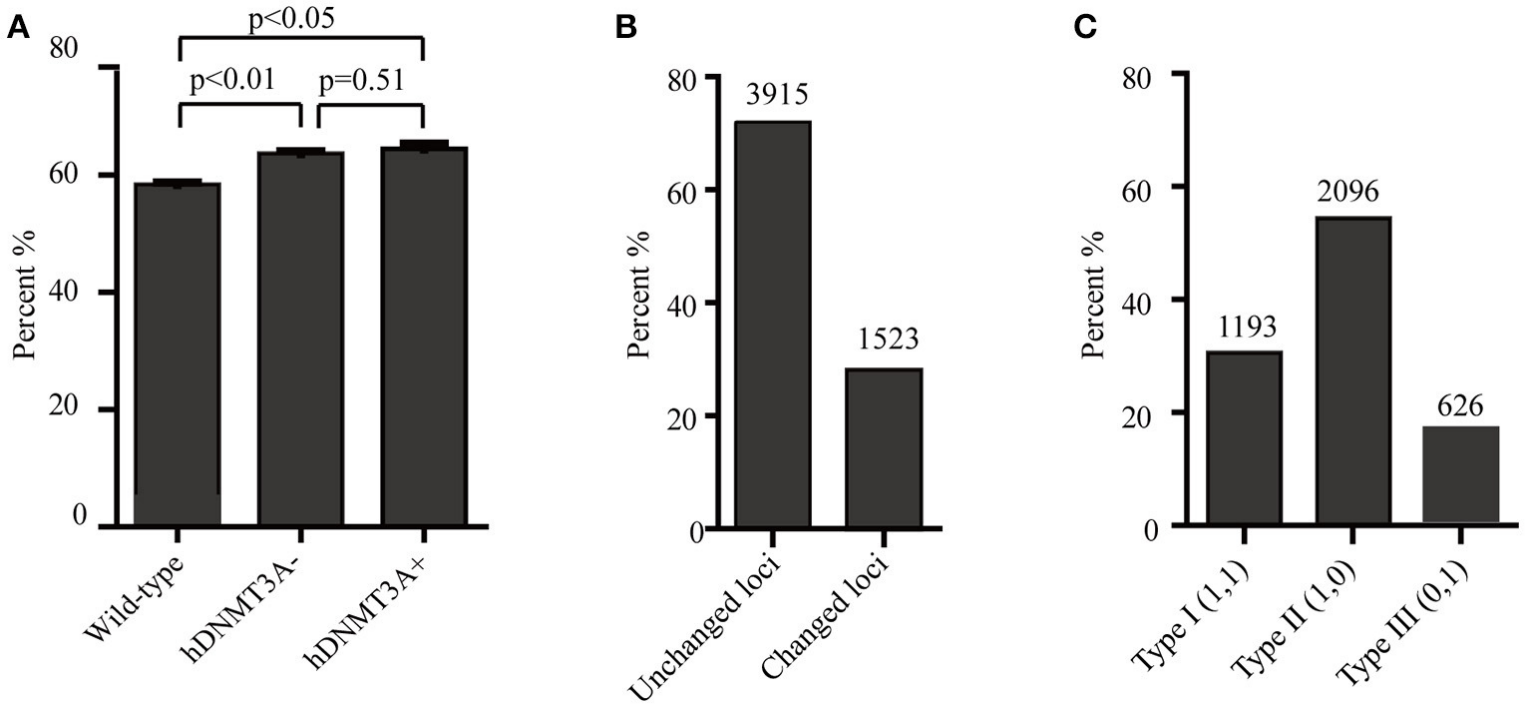
Analysis of the MSAP results. **(A)** DNA methylation ratios of wild-type, *hDNMT3A*^−^, and *hDNMT3A*^+^ rat sperm. **(B)** Analysis of total amplified loci. Unchanged loci represented amplified loci that had no variations in band types among wild-type, *hDNMT3A*^−^ and *hDNMT3A*^+^ rats. Changed loci represented amplified loci that had variations in band types among wild-type, *hDNMT3A*^−^ and *hDNMT3A*^+^ rats. **(C)** The proportions of different band types in unchanged amplified loci.

Since *hDNMT3A* was highly expressed in the transgenic rats, it might participate in the epigenetic reprogramming during embryogenesis and spermatogenesis, and could influence the sperm methylation pattern of the subsequent generations. Here, we performed MSAP to identify the impact of *hDNMT3A* overexpression on DNA methylation patterns of progenies' sperm (F2 generation). A total of 5438 MSAP loci were recorded using 64 selective primer-pair combinations (Supplemental Table 2). Among these loci, the numbers of full-methylated bands (type II) on average were 2,316, 2,527, and 2,545 in wild-type, hDNMT3A^−^ and hDNMT3A^+^ rats, respectively (Table 2). The DNA methylation ratios of wild-type, hDNMT3A^−^ and hDNMT3A^+^ rats were 59.01, 63.62, and 64.40%, respectively (Table 2). DNA methylation ratios were higher than 50% in all tested sperm samples, suggesting highly methylated patterns in rat sperm. In addition, the DNA methylation ratios of wild-type rats were lower than those of both *hDNMT3A*^−^ and *hDNMT3A*^+^ rats, suggesting that *hDNMT3A* overexpression in paternal testis might affect the DNA methylation pattern of the progenies' sperm (Table 2, Figure 3A). The increase in the DNA methylation ratio was due to the increased number of fully methylated loci.

**Table 2:** DNA methylation patterns in sperm of both *hDNMT3A*^−^ and *hDNMT3A*^+^ rats.

**Genotype**	**Wild-type**			**Negative (*hDNMT3A*-)**			**Positive (*hDNMT3A*±)**		
Replications	Rep1	Rep2	Rep3	Rep1	Rep2	Rep3	Rep1	Rep2	Rep3
Type I (1,1)	1,790	1,802	1,926	1,425	1,407	1,505	1,346	1,374	1,503
Type II (1.0)	1,994	2,063	2,092	2,538	2,525	2,519	2,543	2,587	2,505
Type III (0.1)	813	844	840	846	887	860	948	878	853
Type IV (0.0)	841	729	580	629	619	554	601	599	577
All ampli. Loci^*^	5,438	5,438	5,438	5,438	5,438	54,38	5,438	5,438	5,438
Methyl. (%)^**^	52.70	53.38	52.07	64.04	64.22	62.60	65.39	65.31	62.50
Unmethyl. (%)	47.30	46.62	47.93	35.96	35.78	37.40	34.61	34.69	37.50

* The numbers of all selective-amplified fragments that used for further analysis.

** *The percent of methylated loci*.

To investigate the influence of *hDNMT3A* overexpression on sperm DNA methylation pattern, we analyzed the DNA methylation changes among sperm of wild-type, hDNMT3A^−^ and hDNMT3A^+^ rats. In this study, we defined hypermethylation when the amplified bands changed from type I (unmethylation) to type II (full-methylation), and defined hypomethylation when the amplified bands changed from type II to type I (Table 1). In addition, the loss of CmCGG (from type II to type IV, and from type III to type I) was defined as hypomethylation while the emergence of CmCGG (from type I to type III, and from type IV to type II) was considered as hypermethylation (Table 1, Figure 1). Among 5438 totally amplified loci, we found that 71.99% (3,915 of 5,437) loci had no variations in band types among wild-type, hDNMT3A^−^ and hDNMT3A^+^ rats (Figure 3B). Among these unchanged loci, 30.48, 53.53, and 15.99% were type I (unmethylated), type II (fully-methylated) and type III, respectively (Figure 3C). In addition, there were about 28.01% (1523) loci had variations in band types among wild-type, hDNMT3A^−^ and hDNMT3A^+^, i.e., there were some loci had variations in band types between wild-type and hDNMT3A^−^, between wild-type and hDNMT3A^+^, or between hDNMT3A^−^ and hDNMT3A^+^ (Figure 3B). These variations included mutations in DNA sequence and epimutations in DNA methylation.

### *hDNMT3A* overexpression induced genome-wide alterations in DNA methylation

**Figure 4:**
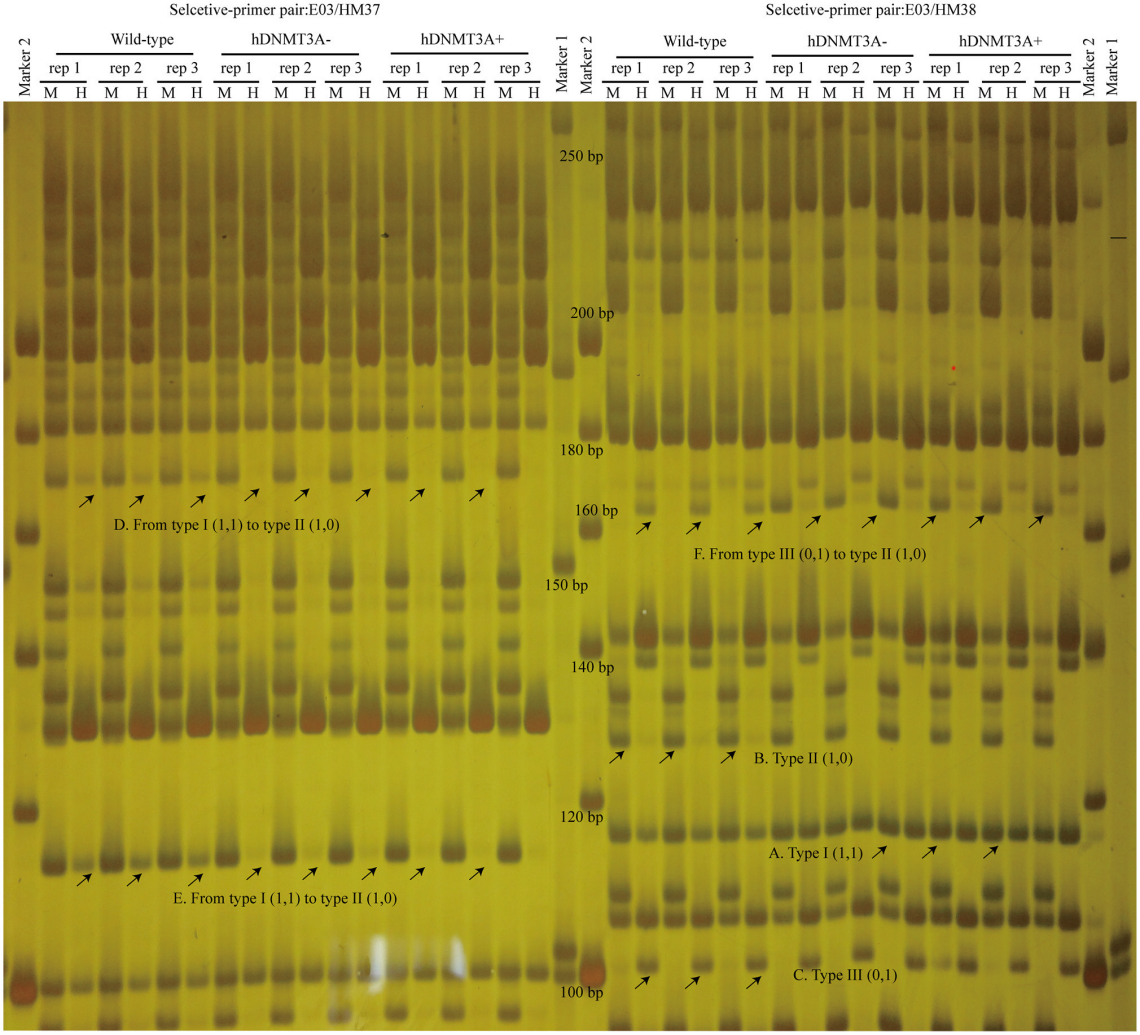
Analyze the differentially amplified loci using polyacrylamide gel electrophoresis and silver staining. M, *Msp* I/*EcoR* I digestion lanes; H, *Hpa* II/*EcoR* I digestion lanes. A: Type I (1,1) bands; B: Type II (1,0) bands; C: Type III (0,1) bands; D: A hypermethylated DAL that the band type tended from Type I to type II. E: another hypermethylated DAL; F: A DNA methylation level unchanged DAL that the band type tended from type III to type II. Arrows indicated corresponding band types.

**Figure 5:**
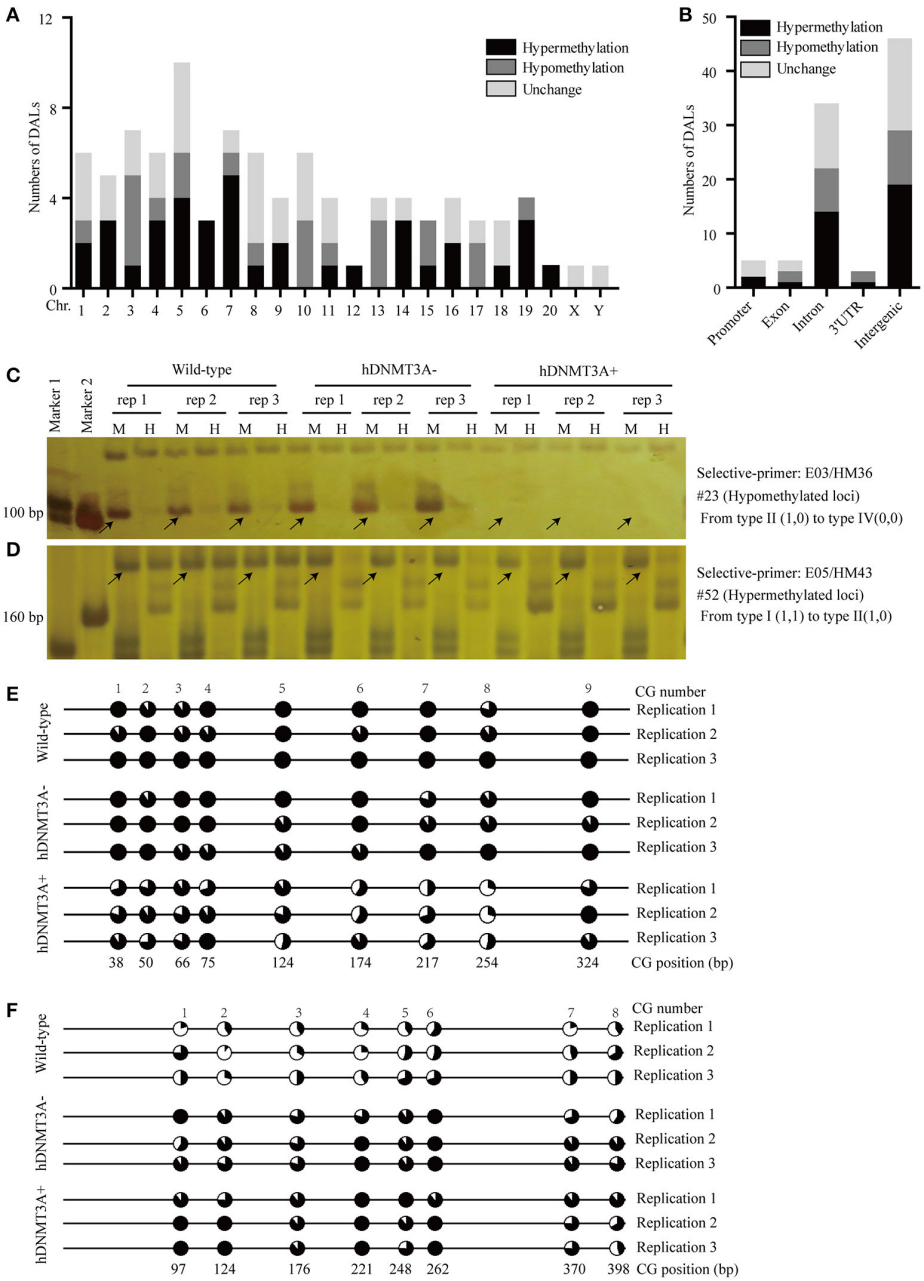
Distribution analysis of the DALs on chromosome level and on genic level. **(A)** Distribution of the hypermethylated, hypomethylated and DNA methylation unchanged DALs on chromosome level. **(B)** Distribution of the hypermethylated, hypomethylated and DNA methylation unchanged DALs on genic level. **(C)** A hypomethylated loci that the band type tended from type II (1,0) to type IV (0,0). **(D)** A hypermethylated loci that the band type tended from type I (1,1) to type II (1,0). **(E)** Using BSP assay to identify the DNA methylation patterns of #23 DAL (Supplemental Table 3) in wild-type, *hDNMT3A*- and *hDNMT3A*+ rats. The eighth CG that located at 254 bp was contained by the *Msp* I/*Hpa* II recognized sequence (5′-C**CG**G-3′). Statistics based on 10 clones per replication. **(F)** Using BSP assay to identify the DNA methylation patterns of #52 DAL (Supplemental Table 3) in wild-type, *hDNMT3A*- and *hDNMT3A*+ rats. The second CG that located at 124 bp was contained by the *Msp* I/*Hpa* II recognized sequence. Arrows indicated corresponding band types.

To investigate the distribution and biological functions of DALs, we performed 6% polyacrylamide gel electrophoresis and silver staining to isolate the differentially methylated fragments (Figure 4, Supplemental Figure 3). Ninety-three randomly selected DALs, including 37 hypermethylated DALs, 22 hypomethylated DALs, and 34 DALs that had no DNA methylation changes, were successfully isolated and re-amplified with appropriate selective primer-pair combinations (Figure 4, Supplemental Figure 3 and Supplemental Table 3). They were cloned into vectors and subsequently sequenced. Distribution analysis revealed that they were widely distributed on all 22 chromosomes of the rat genome (Figure 5A). Hypermethylated DALs were predominantly found in chromosomes 1, 2, 4, 5, 7, 14, 16, and 19, while hypomethylated DALs primarily occurred on chromosomes 3, 10, 13, 15, and 17 (Figure 5A). Analysis on gene levels revealed that these DALs were primarily distributed in the intronic and intergenic regions (Figure 5B, Supplemental Table 3). Interestingly, besides the unchanged DALs, most of the DALs that occurred in promoter regions were hypermethylated while most of the DALs that occurred in exons and 3′UTRs were hypomethylated (Figure 5B). In addition, DALs that occurred in intronic and intergenic regions tended to be hypermethylated (Figure 5B).

To validate the reliability of the MSAP results, we performed BSP assays to identify the DNA methylation variations of two MSAP loci, including one hypomethylated loci (#23) and one hypermethylated loci (#52) (Figures 5C,D, Supplemental Table 3). The results revealed that the #23 loci was hypomethylated in *hDNMT3A*^+^ rats (Figure 5E, Supplemental Table 4), and the #52 loci was hypermethylated in both *hDNMT3A*^−^ and *hDNMT3A*^+^ rats (Figure 5F, Supplemental Table 4). These DNA methylation variations were consistent with the MSAP results, indicating that the MSAP method was reliable.

### *hDNMT3A* overexpression induced intergenerational inheritance of DNA methylation changes

**Figure 6:**
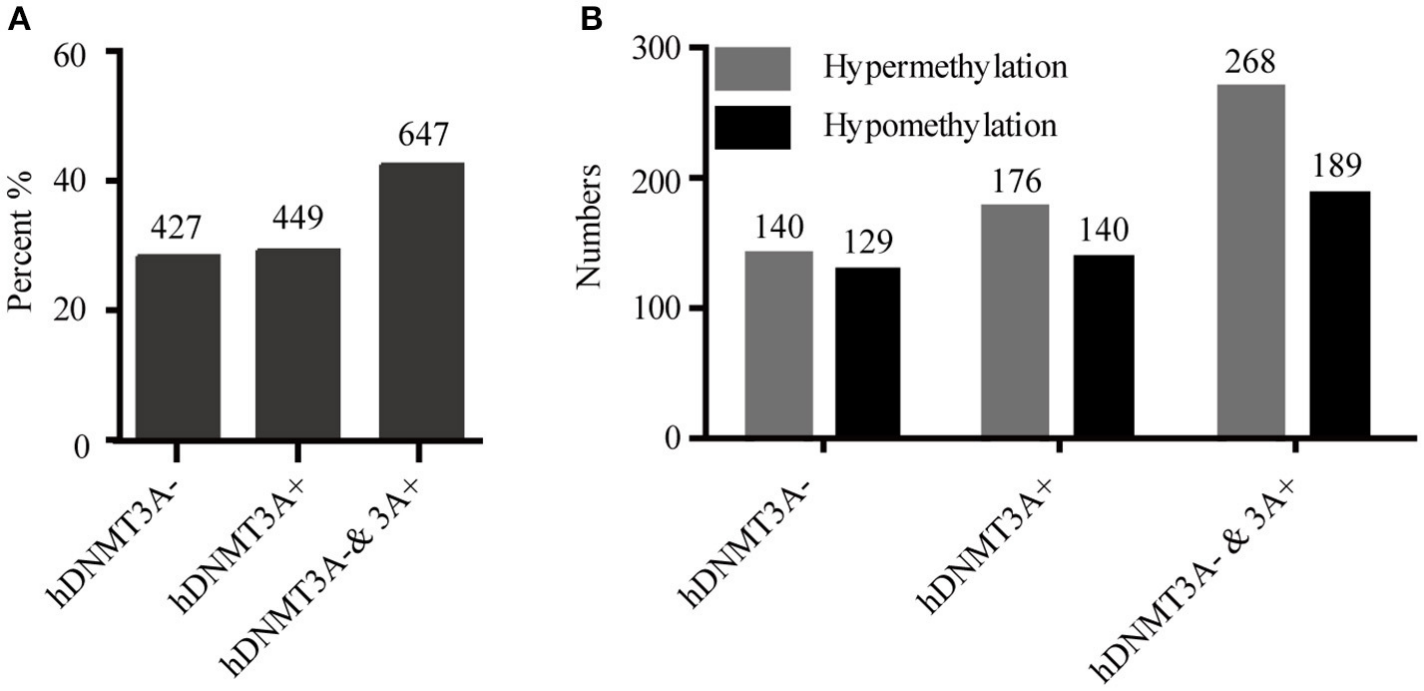
Intergenerational inheritance of differentially methylated loci. **(A)** The distribution of total DALs. **(B)** The distribution of DMLs.

To determine whether the DNA methylation changes induced by *hDNMT3A* overexpression in paternal testis could be transmitted to the progeny, we analyzed the status of these 1,523 in both hDNMT3A^−^ and hDNMT3A^+^ rat sperm (Table 3). Among the 1,523 DALs, 28.01% (427) only occurred between hDNMT3A^−^ and wild-type rats while 29.48% (449) only occurred between hDNMT3A^+^ and wild-type rats (Table 3). The remaining 42.28% (647) loci had the same band types in both hDNMT3A^−^ and hDNMT3A^+^ progenies distinguishing from wild-type rats, suggesting that these mutations and epimutations were transmitted from paternal genome to both genotype-positive and negative offspring (Figure 6A).

**Table 3:** Distribution of DALs in genotype-positive and -negative transgenic rats.

**Changes in band types**	**Only in *hDNMT3A*^−^**	**Only in *hDNMT3A*^+^**	**In both *hDNMT3A*- and *hDNMT3A*^+^**
Type IV (0,0) → Type I (1,1)	5	8	22
Type IV (0,0) → Type II (1,0)	66	95	133
Type IV (0,0) → Type III (0,1)	59	63	83
Type I (1,1) → Type IV (0,0)	9	7	21
Type I (1,1) → Type II (1,0)	53	53	111
Type I (1,1) → Type III (0,1)	21	28	24
Type II (1,0) → Type IV (0,0)	73	59	113
Type II (1,0) → Type I (1,1)	40	61	43
Type II (1,0) → Type III (0,1)	0	0	0
Type III (0,1) → Type IV (0,0)	85	55	63
Type III (0,1) → Type I (1,1)	16	20	33
Type III (0,1) → Type II (1,0)	0	0	1
Total	427	449	647

*All three replicates had same band types*.

Among these 1523 DALs, 1042 (68.42%) DALs had DNA methylation variations in hDNMT3A^−^ and/or hDNMT3A^+^ rats when compared with wild-types (i.e., 1042 DMLs) (Table 3). Among the 647 DALs that occurred in both *hDNMT3A*^−^ and *hDNMT3A*^+^ rats, there were 268 hypermethylated and 189 hypomethylated DMLs (Table 3, Figure 6B), accounting for 41.42% and 29.21%, respectively. These DMLs accounted for 34.26% of total DMLs. They were transmitted from paternal epigenome and kept stable in genotype-negative transgenic offspring, indicating that DNA methylation changes in these loci were induced by *hDNMT3A* overexpression in paternal testis and could be transmitted to offspring in the absence of hDNM3A. In other words, *hDNMT3A* overexpression in paternal testis could induce intergenerational inheritance of DNA methylation changes even in the absence of hDNM3A. These intergenerational inheritance DMLs accounted for 43.86% of total DMLs. In addition, among the 449 DALs that only occurred in hDNMT3A^+^, there were 176 hypermethylated and 140 hypomethylated DMLs (Figure 6B). We inferred that these DMLs depended on *hDNMT3A* overexpression as they were not changed in DNA methylation status in *hDNMT3A*^−^ rat sperm. Among the 427 DALs that only occurred in hDNMT3A^−^, there were 140 hypermethylated and 129 hypomethylated DMLs (Figure 6B). We inferred that these DMLs responded to *hDNMT3A* removal.

### DML-related genes involved in a wide range of functions

Gene ontology analysis was performed to analyze the functions of genes that related to DMLs. There were 2, 3, 3, and 22 genes related to promoter DML, exon DML, 3′UTR DML and intron DML, respectively (Supplemental Table 3). GO analysis revealed that among these 30 genes, 24 genes mapped to the reference list of *Rattus norvegicus*. These genes were involved in a wide range of functions. Both two genes that related to promoter DMLs were hypermethylated. One encoded an intracellular Ca^2+^ channel protein (*Mcoln1*), which was required for sarcolemma repair to prevent muscular dystrophy. Another encoded a phospholipid-binding protein *Sestd1* which negatively regulates dendritic spine density by interfering with Rac1-Trio8 signaling pathway (Supplemental Table 3). All the 3 genes (*Nup153, Zfp111, Wdfy3*) that related to exon DMLs were hypomethylated. *Nup153 was* a nuclear pore complex protein, it might act to gate transcribable genes to nuclear pore complexes. Depletion of Nup153 in mouse embryonic stem cells (mESCs) causes the derepression of developmental genes and induction of early differentiation. *Zfp111* Had transcriptional repressor activity, and it might act as a transcriptional repressor in oligodendrocytes of the CNS. Loss of *Wdfy3* led to a regionally enlarged cerebral cortex resembling early brain overgrowth described in many children on the autism spectrum (Supplemental Table 3). Among the 3 genes that related to 3′UTR DML, one was hypermethylated and encoded an E3 ubiquitin-protein ligase that contained a RING-H2 finger (*Rnf103*). The other 2 genes were hypomethylated and encoded a pseudogene (*RGD1561310*) and stefin A2-like 2 protein (*Stfa2l2*), respectively (Supplemental Table 3). In addition, 22 genes that related to intron DMLs contained 14 hypermethylated and 8 hypomethylated genes. GO analysis revealed that these genes had binding, catalytic, transporter, receptor and signal transducer activities, and they paricipated in development, reproduction, metabolic and other processes. For examples, *Pard3* was related to #23 hypomethylated DML, it promoted the interaction between PP1A and LATS1 to induce LATS1 dephosphorylation and inactivation, therefore leading to dephosphorylation and activation of TAZ (Figures 5C,E, Supplemental Table 3); another gene *Cdh13* was related to #52 hypermethylated DML. It involved in cell growth regulation as a cell adhesion molecule (Figures 5D,F, Supplemental Table 3).

## Discussion

Increasing evidence has suggested that parental environmental exposure, such as toxicants and stress, could influence the development of future generations [14–16]. This “environmental information” was transmitted from parents to offspring through the germline. DNA methylation was the major focus of studies of paternal epigenetic inheritance, but only minor changes in sperm have been related to transmission of environmentally induced traits (Carone et al., 2010; Lambrot et al., 2013; Dias and Ressler, 2014). Nevertheless, these inheritable DNA methylation changes were passively induced by environmental factors, and the underlying mechanisms of how they overrode the reprogramming process for transgenerational inheritance was still unclear. DNA methyltransferases, including DNMT1, DNMT3A, DNMT3B, DNMT3C, and the cofactor DNMT3L, play important roles in the establishment and maintenance of DNA methylation patterns during spermatogenesis and embryogenesis, especially in the epigenetic reprogramming process (Law and Jacobsen, 2010; Barau et al., 2016). In this study, we actively changed the DNA methylation pattern of rat sperm by altering the activity of DNA methyltransferase in the testis. We found that active DNA methylation changes in sperm that were induced by *hDNMT3A* overexpression in testis could also be intergenerationally inherited by offspring in the absence of *hDNMT3A* transmission. This study provided evidence for the transmission of epigenetic variations that were actively induced by endogenous factors, and the inheritance of epigenetic variations was independent of the persistence of these endogenous factors. We hypothesized that the inheritance of the environmental factor-induced DNA methylation changes via the gametes would be closely related to the activity of DNA methyltransferases and DNA demethylases during the epigenetic reprogramming process. In addition, epigenetic changes in mammals can arise sporadically (actively changes) and can be induced by the environment, such as toxins, nutrition and stress (passively changes). If a pregnant female mouse (F0) was exposed to an experimental factor, the fetus can be affected *in utero* (F1), as can the germline of the fetus (the future F2). These are considered to be parental effects, leading to intergenerational epigenetic inheritance. Only F3 individuals can be considered as true transgenerational inheritance (Heard and Martienssen, 2014). In the case of males in which an epigenetic change is induced, the individual (F0) and his germline (future F1) are exposed, the F1 is thus considered as intergenerational. Only F2 and subsequent generations can be considered for evidence of transgenerational inheritance (Heard and Martienssen, 2014). In this study, the question that how many generations could stably inherit the *hDNMT3A* overexpression-induced DNA methylation changes in the absence of *hDNMT3A* requires further research. In addition, whether the overexpression of other DNA methyltransferases, such as DNMT1, DNMT3B, DNMT3C, and DNMT3L, could induce transgenerational inheritance of DNA methylation changes in mammal sperm remains to be elucidated, as these proteins are also involved in establishing the DNA methylation pattern of mature sperm (He et al., 2011; Barau et al., 2016).

Abnormal expression of DNA methyltransferases was reported to induce dynamic DNA methylation changes, and disturbance of intrinsic DNA methylation patterns might lead to diseases in mammals or even lethality (Bergman and Cedar, 2013; Fu et al., 2016). In this study, we found that abnormal expression of *hDNMT3A* in rat testis resulted in widespread changes in DNA methylation in mature sperm, but we did not observe side effects of these DNA methylation changes, as the reproductive rat and embryo development of the progenies appeared to be normal. This could be due to both the rectification by enzymes that related to demethylation and embryonic tolerance to a certain extent of variations in DNA methylation. Our results showed that there were also high proportions of intergenerational-inherited hypomethylated DMLs in the offspring. We hypothesized that genes related to these hypomethylated DMLs would respond to or act instead of the genes related to abnormal hypermethylated DMLs. In addition, overexpression of *DNMT1, 3A*, and *3B* was correlated with tumorigenesis and other diseases (Qu et al., 2010; Rahman et al., 2010; Siddiqui et al., 2016), and *DNMT3A* expression was significantly associated with the prognosis of gastric and hepatocellular carcinomas in human (Oh et al., 2007; Cao et al., 2014). More research is needed to confirm whether the DNA methylation patterns changed in liver and brain, in which the *hDNMT3A* is also highly expressed, and whether the altered DNA methylation patterns affects their function.

In addition to DNA methylation, histone modifications and non-coding RNA could also mediate the inheritance of acquired traits that were induced by environmental factors in mammals. For example, a low-protein diet induced a consistent decrease in H3K27me3 in mouse sperm, and these histone modifications cooperating with DNA methylation changes mediated the inheritance of low-protein diet-induced phenotypes to offspring (Carone et al., 2010). Sperm tRNA-derived small RNAs (tsRNAs) mediated the inheritance of phenotypes that were induced by a high-fat diet in male mice, indicating sperm tsRNAs represent another type of paternal epigenetic factor that mediate intergenerational inheritance of diet-induced metabolic disorders (Chen et al., 2016). In addition to environmental factor-induced DNA methylation changes, these changes in histone modifications and non-coding RNAs were also changed passively. We hypothesized that actively disturbed patterns of intrinsic histone modifications and RNA expression changed the activity of corresponding endogenous factors, such as methyltransferases, demethylases, acetyltransferases and deacetylases for histone modifications and RNA synthetases and covalent modification enzymes for tsRNAs, and could induce transgenerational inheritance of the acquired phenotypes. A study showing overexpression of hKDM1A during spermatogenesis validated this assumption (Siklenka et al., 2015). Conditional overexpression of the hKDM1A in gonads of male mice reduced the H3K4me2 in their mature sperm, resulting in severely impaired development and survival of offspring, and the phenotype and altered histone modification pattern could be transmitted at least four generations (Siklenka et al., 2015).

In conclusion, our results revealed that active changes in DNA methylation in rat sperm through overexpression of *hDNMT3A* in paternal testis could be intergenerationally inherited to offspring without the transmission of *hDNMT3A*. Most of the inheritable DNA methylation changes were hypermethylated, but a considerable proportion of hypomethylated DMLs could also be inherited. This study provided evidence for the transmission of active endogenous-factors-induced epigenetic variations. More research is required to elucidate the functional links among environmental factors, epigenetic modifiers, the composition of the sperm epigenome, and the consequent altered phenotypes in offspring.

## Author contributions

FS, XH, and YM designed the work, XZ and ZHL wrote the manuscript, XZ performed MSAP assay, ZHL performed the BSP assay, GW performed the qPCR assay to analyzed the expression pattern of transgene, AL performed rat sperm isolation and DNA extraction, HW performed the qPCR assay to analyzed the copy numbers of transgenic rats, YD performed issue DNA extraction and analyzed the MSAP data that detected by polyacrylamide gel electrophoresis, ZZL re-amplified the differentially amplified loci. XH analyzed the qPCR data and MSAP data that detected by capillary electrophoresis, YM and XC performed the zygote microinjection of bacterial artificial chromosome.

### Conflict of interest statement

The authors declare that the research was conducted in the absence of any commercial or financial relationships that could be construed as a potential conflict of interest.

## Supplementary material

Supplemental Figure 1 0.5% agarose gels to ensure that the genomic DNA was completely digested. M represent *EcoR* I/*Msp* I lane, H represent *EcoR* I/*Hpa* II lane. A negative control which the genomic DNA was incubated but without enzymes was set.

Supplemental Figure 2 Using capillary electrophoresis (CE) to analyze the product of selective amplification. Only the bands that signal height (Y axis) was more than 1,000 and the length of fragments (X axis) were between 50 and 500 bp were used for further analysis. **(A)** A sample was tested by CE, one lane was digested by *Msp* I/*EcoR* I and another lane was digested by *Hpa* II/*EcoR* I. Type I represented unmethylated loci (these loci could be digested by both *Msp* I and *Hpa* II); Type II represented full-methylated loci, i.e., the two strands of DNA were methylated in the recognized CG dinucletide (these loci could be digested only by *Msp* I); Type III represented by hemi-methylated loci, i.e., only one of the two strands of DNA was methylated in the recognized CG dinucletide (these loci could be digested only by *Hpa* II). **(B)** The model of different band types: a. full-methylated loci (type II); b. unmethylated loci (type I); c. hemi-methylated loci (type III); d. hyper-methylated loci (type IV, the loci could be detected by comparing with other samples). Arrows indicated corresponding band types.

Supplemental Figure 3 Using polyacrylamide gel electrophoresis (PAGE) to analyze the product of selective amplification, and to recover differentially amplified loci (DALs). Only the differentially amplified fragments that the length between 50 and 500 bp were reamplified and used for further analysis. A: type I (1,1), unmethylated loci; B: type II (1,0), full-methylated loci; C: type III (0,1); D: type IV (0,0). E: a DAL that the band type tended from type IV (0,0) to type II (1,0). Arrows indicated corresponding band types.

Supplemental Table 1 8 PCR primer-pairs were specific for the RP11-159D24 sequence to detect positive transgenic rats.

Supplemental Table 2 Primers used for MSAP analysis.

Supplemental Table 3 Information about 93 DALs which were reamplified and sequenced.

Supplemental Table 4 Bisulfite sequencing PCR (BSP) data.

## Abbreviations

MSAP: Methylation sensitive amplification polymorphisms
BSP: Bisulfite sequencing PCR
DAL: Differentially amplified loci
DML: Differentially methylated loci.
