# Supplementary figures and images for: Overexpression of Human-Derived DNMT3A Induced Intergenerational Inheritance of Active DNA Methylation Changes in Rat Sperm
Source: Front Genet. 2017 Dec 12;8:207. doi: 10.3389/fgene.2017.00207 (PMC5733082; doi:10.3389/fgene.2017.00207)

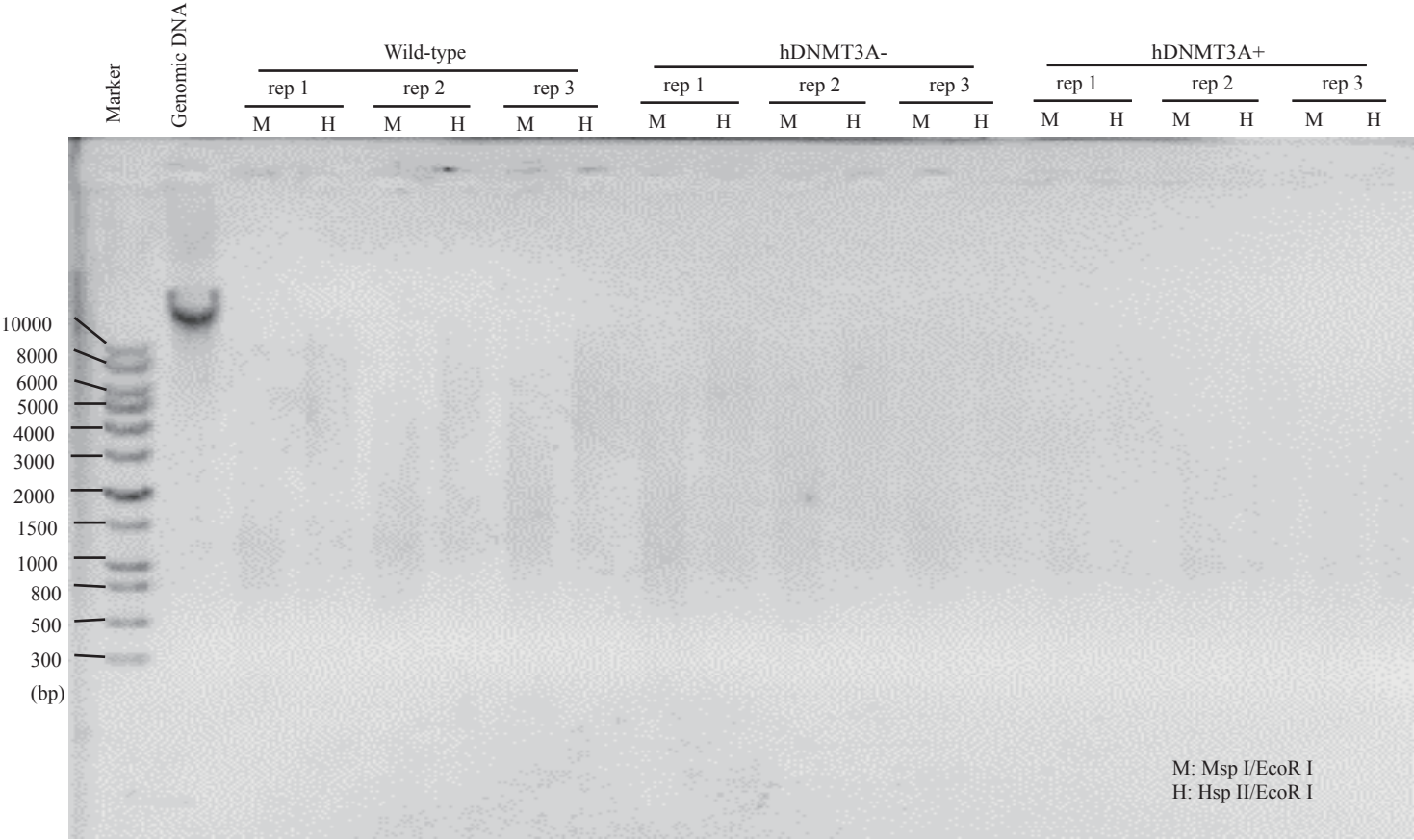

Supplement: Supplemental Figure 1 — 0.5% agarose gels to ensure that the genomic DNA was completely digested. M represent EcoR I/Msp I lane, H represent EcoR I/Hpa II lane. A negative control which the genomic DNA was incubated but without enzymes was set. [file Image1.PDF]

A

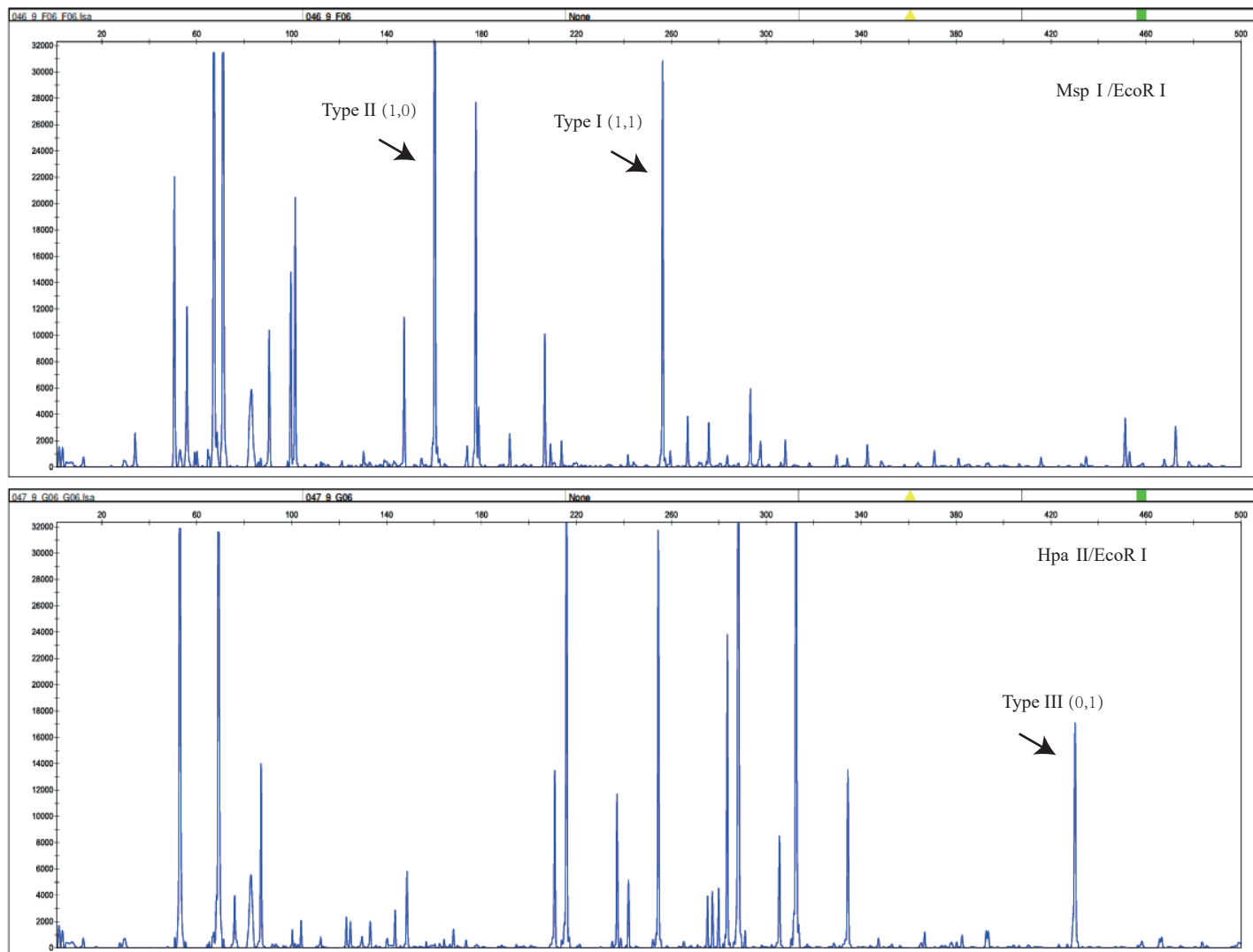

B

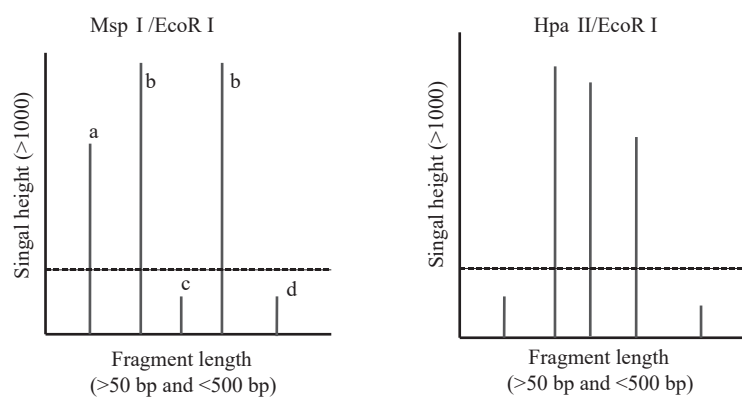

Supplement: Supplemental Figure 2 — Using capillary electrophoresis (CE) to analyze the product of selective amplification. Only the bands that signal height (Y axis) was more than 1,000 and the length of fragments (X axis) were between 50 and 500 bp were used for further analysis. (A) A sample was tested by CE, one lane was digested by Msp I/EcoR I and another lane was digested by Hpa II/EcoR I. Type I represented unmethylated loci (these loci could be digested by both Msp I and Hpa II); Type II represented full-methylated loci, i.e., the two strands of DNA were methylated in the recognized CG dinucletide (these loci could be digested only by Msp I); Type III represented by hemi-methylated loci, i.e., only one of the two strands of DNA was methylated in the recognized CG dinucletide (these loci could be digested only by Hpa II). (B) The model of different band types: a. full-methylated loci (type II); b. unmethylated loci (type I); c. hemi-methylated loci (type III); d. hyper-methylated loci (type IV, the loci could be detected by comparing with other samples). Arrows indicated corresponding band types. [file Image2.PDF]

Selctive-primer pair:E04/HM39

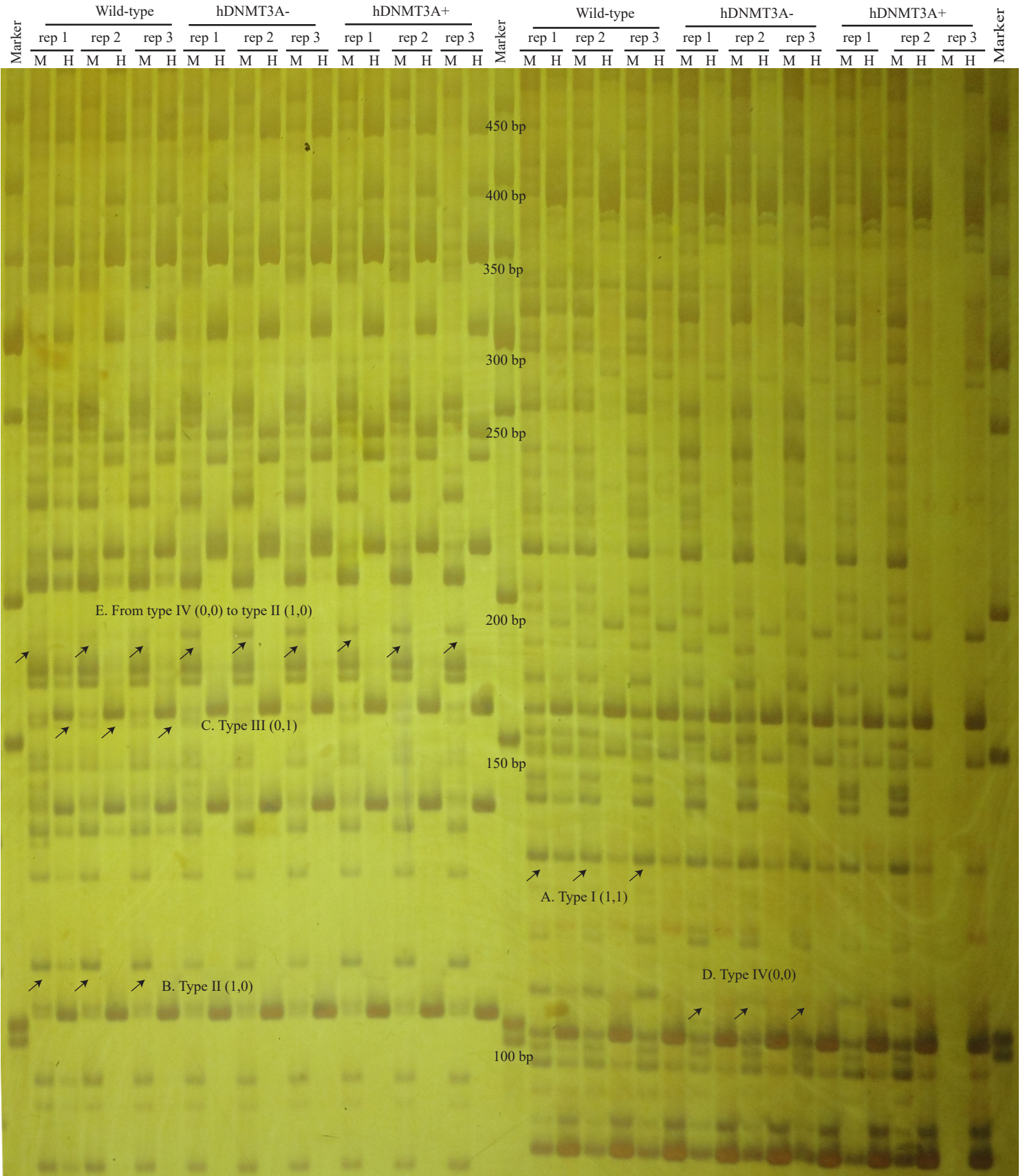

Supplement: Supplemental Figure 3 — Using polyacrylamide gel electrophoresis (PAGE) to analyze the product of selective amplification, and to recover differentially amplified loci (DALs). Only the differentially amplified fragments that the length between 50 and 500 bp were reamplified and used for further analysis. A: type I (1,1), unmethylated loci; B: type II (1,0), full-methylated loci; C: type III (0,1); D: type IV (0,0). E: a DAL that the band type tended from type IV (0,0) to type II (1,0). Arrows indicated corresponding band types. [file Image3.PDF]
